# Impact of Dupilumab Therapy on Skin Barrier Function in Patients With Moderate‐to‐Severe Atopic Dermatitis: A Phase IV, Non‐Randomized, Open‐Label, Parallel‐Group Clinical Study

**DOI:** 10.1002/mco2.71091

**Published:** 2026-10-25

**Authors:** Taoming Liu, Shuting Chen, Weijia Zhao, Dake Di, Jiao Liu, Yuyang Tian, Yurong Luo, Yingping Xu, Ruzeng Xue, Li Ye, Xiaoping Pei, Bihui Yu, Yu Wang, Jiao Zhang, Xiaoling Yu, Wei Wen, Liyan Yuan, Suyun Ji, Claire Hu, Dan Shen, Yiqing Guo, Yunsheng Liang

**Affiliations:** ^1^ Hunan Key Laboratory of Medical Epigenomics The Second Xiangya Hospital of Central South University Changsha China; ^2^ Department of Dermatology Dermatology Hospital Southern Medical University Guangzhou China; ^3^ The First School of Clinical Medicine Southern Medical University Guangzhou China; ^4^ Department of Traditional Chinese Medicine Dermatology Dermatology Hospital Southern Medical University Guangzhou China; ^5^ Dermatology Hospital Institute of Dermatology and Venereology Southern Medical University Guangzhou China; ^6^ Evaluation Center for Skin Care Product Efficacy Dermatology Hospital Southern Medical University Guangzhou China; ^7^ Medical Affairs Sanofi Shanghai China; ^8^ Medical Affairs Sanofi Beijing China; ^9^ Medical Affairs Sanofi Guangzhou China; ^10^ Department of Dermatology Second Xiangya Hospital Central South University Changsha China

**Keywords:** atopic dermatitis, dupilumab, lipidomics, proteomics, skin barrier function

## Abstract

Dupilumab improves skin barrier function in patients with atopic dermatitis (AD), but data for Chinese patients are limited. This 16‐week, prospective, exploratory study investigated the impact of dupilumab on skin barrier function in Chinese patients with moderate‐to‐severe AD. Forty‐four participants (24 patients with AD and 20 healthy volunteers) were enrolled. Patients with AD received dupilumab treatment every 2 weeks and were followed up to Week 16. Tape‐stripped stratum corneum samples were collected from participants at seven specific time points for proteomic and lipidomic profiling, together with transepidermal water loss (TEWL) measurement. At Week 16, TEWL after five tape strips was significantly reduced versus baseline (adjusted *p* = 0.0006). Proteomics revealed significant upregulation of sphingolipid and other lipid metabolism pathways within the first 2 weeks, coinciding with early TEWL improvement; keratinocyte differentiation and keratinization pathways were enriched from Week 8 through Week 16. Lipidomics revealed that specific dysregulated ceramides—CER(d16:0/16:0), CER(d16:0/20:3), CER(t20:1/23:0), and CER(t20:1/24:1)—showed significant changes by Weeks 2–8 and completely normalized by Week 16. Dupilumab improved skin barrier function in Chinese patients with moderate‐to‐severe AD, accompanied by dynamic molecular changes; these correlative findings require functional validation to establish causality.

Trial Registration: ClinicalTrials.gov NCT05624112.

## Graphical abstract

This 16‑week study assessed impact of dupilumab on skin barrier function in patients with moderate‑to‑severe atopic dermatitis (AD). Tape‑stripped stratum corneum samples for multi‑omic profiling, alongside transepidermal water loss measurements, were collected from lesional/non‑lesional AD skin and healthy controls at seven time points. Dupilumab improved skin barrier function and correlated with upregulated lipid metabolism, enhanced keratinocyte differentiation, and reduced serine peptidase expression in lesional skin. These correlative findings provide temporal molecular insights but require functional validation.

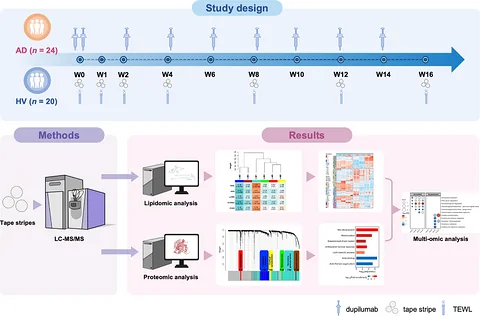

## 1 Introduction

Atopic dermatitis (AD) is a chronic inflammatory skin disorder affecting 15%–20% of children and up to 10% of adults [1]. Its clinical manifestations—ranging from papules, edema, and scaling to hyperpigmentation, excoriations, bleeding, or hemorrhagic crusts—can substantially impair health‐related quality of life (HRQoL) [1, 2]. Although several conventional treatments exist for moderate‐to‐severe AD, including systemic corticosteroids, narrow‐band UVB (NB‐UVB) phototherapy, and azathioprine. However, these options are associated with various adverse effects, and NB‑UVB in particular may be inconvenient to administer [3].

Dupilumab, a fully human monoclonal antibody targeting the alpha subunit of the interleukin (IL)‐4 receptor, inhibits IL‐4 and IL‐13 signaling [4]. The National Medical Products Administration (NMPA) of China has approved dupilumab for individuals with moderate‐to‐severe AD (aged ≥ 6 months), adult prurigo nodularis, adult bullous pemphigoid, asthma (aged ≥ 6 years), and chronic obstructive pulmonary disease (COPD) maintenance therapy in adults [5].

The pathogenesis of AD involves complex interactions among genetic and environmental factors, microbial abnormalities, Type 2 immune dysregulation, and skin barrier dysfunction [6, 7]. A defective epidermal permeability barrier facilitates allergen and microbial penetration, triggering Type 2 cytokine release [8]. In AD lesions, the expression of numerous genes and abundance of proteins governing terminal keratinocyte differentiation and cornification are impaired [9]. Ultrastructural analyses have revealed decreased corneocyte compaction, lower levels of intercellular lipids, disruption of the cornified envelope (CE), and degradation of corneodesmosomes [10]. In AD lesions, lipid alterations include reduced ultra‐long‐chain ceramides (CERs) and shortened free fatty acids (FAs) chain length, as well as increased short‐chain CERs and cholesterol‐3‐sulfate [11]. Transepidermal water loss (TEWL), an indicator of epidermal permeability barrier function, correlates positively with clinical AD severity [12, 13]. Skin tape stripping (STS) is a noninvasive method to collect skin samples for proteomic, lipidomic, and transcriptomic analyses [14, 15, 16, 17, 18]. Moreover, skin barrier integrity can be assessed by combining TEWL measurements and STS [19].

Dupilumab reduces Type 2 inflammation and improves skin barrier function [20]. However, its specific effects on skin barrier function in Chinese patients with AD during therapy remain poorly characterized. Thus, we conducted a 16‐week, open‐label, exploratory study integrating longitudinal proteomic and lipidomic profiling with TEWL measurements in Chinese patients with moderate‐to‐severe AD during dupilumab treatment. Our findings revealed distinct temporal patterns of lipidomic and proteomic changes that paralleled TEWL recovery. These findings provide novel insights into the skin barrier response to IL‐4Rα blockade and highlight that the observed associations are correlative and require functional validation.

## 2 Results

### 2.1 Participants

Between November 25, 2022, and January 25, 2024, 44 participants were enrolled: 24 patients with moderate‐to‐severe AD and 20 healthy volunteers (controls; Figure 1 and Figure S1). Of these, 43 completed the study. One patient with AD voluntarily withdrew from the study at Week 3 after discontinuing dupilumab administration. This patient was included in the clinical analyses until withdrawal but was excluded from the multi‐omic analyses because of insufficient serial samples. Baseline demographics were comparable between the participant groups (Table 1).

**FIGURE 1:**
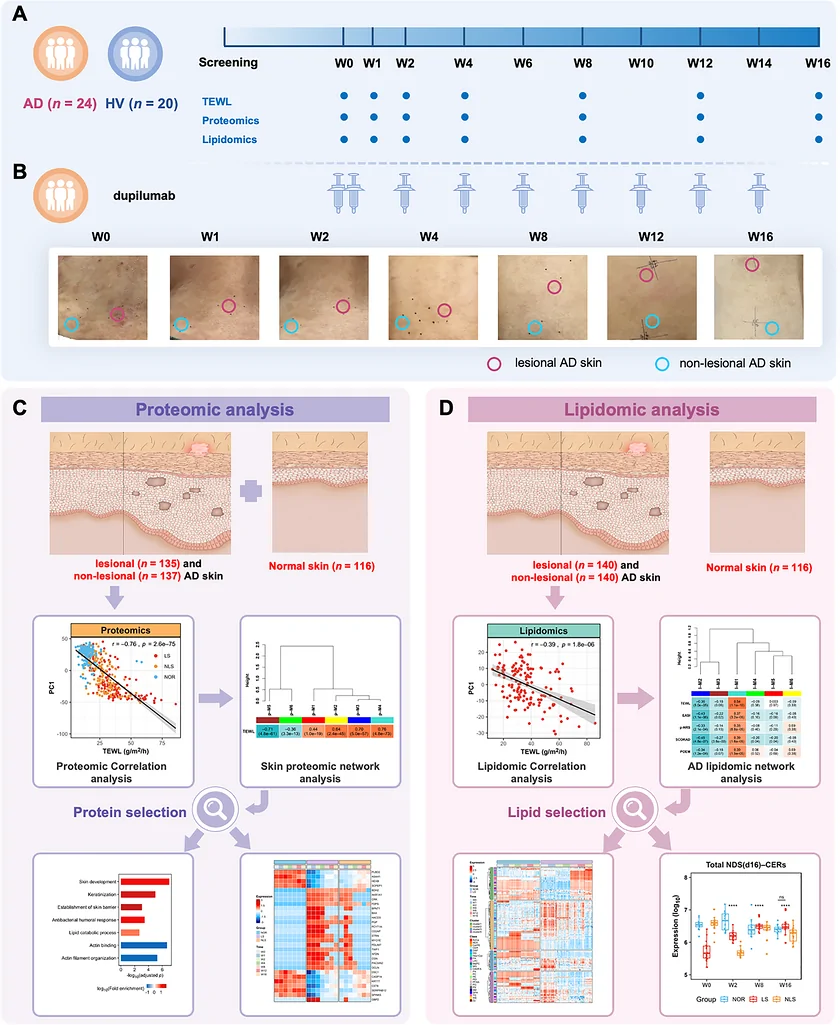
Study design and multi‐omic workflow. This study evaluated skin barrier function in patients with moderate‐to‐severe AD over 16 weeks of dupilumab treatment. (A and B) Clinical study design and sampling scheme. Tape‐stripped skin samples were collected from lesional skin and non‐lesional skin of patients with AD (*n* = 23), and from normal skin of HV (*n* = 20) for proteomics and lipidomics. Samples were obtained at baseline (W0) and Weeks 1, 2, 4, 8, 12, and 16 during dupilumab treatment. (C and D) Analytical workflows for proteomics and lipidomics, illustrating the procedures from sample collection and LC‐MS/MS data acquisition to bioinformatics and statistical analyses. AD, atopic dermatitis; HV, healthy volunteers; W, week.

**TABLE 1:** Demographics and clinical characteristics of study participants at baseline.

	Patients with AD *n* = 24	Healthy volunteers *n* = 20
Age, years		
Mean ± SD	23.3 ± 9.0	24.5 ± 6.5
Median (range)	22.0 (12–47)	24.5 (14–37)
Age category, *n* (%)		
≥ 12 to < 18 years	7 (29.2)	4 (20.0)
≥ 18 years	17 (70.8)	16 (80.0)
Sex, *n* (%)		
Male	14 (58.3)	12 (60.0)
Female	10 (41.7)	8 (40.0)
Height, cm		
Mean ± SD	166.7 ± 6.9	166.5 ± 9.1
Median (range)	166.5 (153.0–178.0)	166.0 (147.0–180.0)
Weight category, *n* (%)		
< 60 kg	13 (54.2)	8 (40.0)
≥ 60 kg	11 (45.8)	12 (60.0)
Weight, kg		
Mean ± SD	61.9 ± 13.8	60.1 ± 13.0
Median (range)	58.5 (44.5–97.0)	62.75 (35.5–95.0)
BMI, kg/m^2^		
Mean ± SD	22.1 ± 3.8	21.6, ± 3.7
Median (range)	22.0 (16.5–31.3)	20.9 (14.5–29.3)
EASI score		
Mean ± SD	29.7 ± 9.3	
Median (IQR)	29.0 (21.8–37.8)	
Min, max	15.80, 49.20	
IGA, *n* (%)		
IGA 3	9 (37.5)	
IGA 4	15 (62.5)	
SCORAD score		
Mean ± SD	64.3 ± 11.59	
Median (IQR)	66.0 (54.0–72.0)	
Min, max	48.0, 91.0	
p‐NRS score		
Mean ± SD	5.6 ± 1.9	
Median (IQR)	5.0 (4.0–7.0)	
Min, max	3.0, 9.0	
DLQI/CDLQI score		
Mean ± SD	12.5 ± 5.24	
Median (IQR)	13.0 (9.0–17.0)	
Min, max	4.0, 25.0	

Abbreviations: AD, atopic dermatitis; BMI, body mass index; CDLQI, Children's Dermatology Life Quality Index; DLQI, Dermatology Life Quality Index; EASI, Eczema Area and Severity Index; IGA, investigator's global assessment; IQR, interquartile range; p‐NRS, pruritus Numerical Rating Scale; SCORAD, SCORing Atopic Dermatitis; SD, standard deviation.

### 2.2 TEWL

At baseline, AD lesions displayed significantly higher TEWL compared with non‐lesional AD skin (mean difference, 23.0; 95% confidence interval [CI], 13.5 to 32.5; adjusted *p* < 0.0001) and normal skin (mean difference, 38.5; 95% CI, 31.0–46.0; adjusted *p* < 0.0001). The change in TEWL after five STS procedures was significantly reduced by Week 2 of dupilumab treatment (adjusted *p* = 0.0036 vs. baseline); this reduction was sustained through Week 16 (adjusted *p* = 0.0006 vs. baseline; Figure 2A). The median (interquartile range [IQR]) TEWL of AD lesions decreased from 52.1 (38.3, 59.7) at baseline to 39.2 (34.5, 53.6) at Week 2 (mean difference, 8.9; 95% CI, 0.1–17.8; adjusted *p* = 0.048), and further decreased to 28.3 (19.5, 32.9) at Week 16 (mean difference, 23.7; 95% CI, 14.3–33.1; adjusted *p* < 0.0001; Figure 2B). At Week 16, TEWL values in lesional and non‐lesional AD skin were comparable (mean difference, 4.2; 95% CI, −2.1 to 10.5; adjusted *p* = 0.247), although they remained significantly higher than those of normal skin (mean difference, 12.3; 95% CI, 7.0–17.6; adjusted *p* < 0.0001).

**FIGURE 2:**
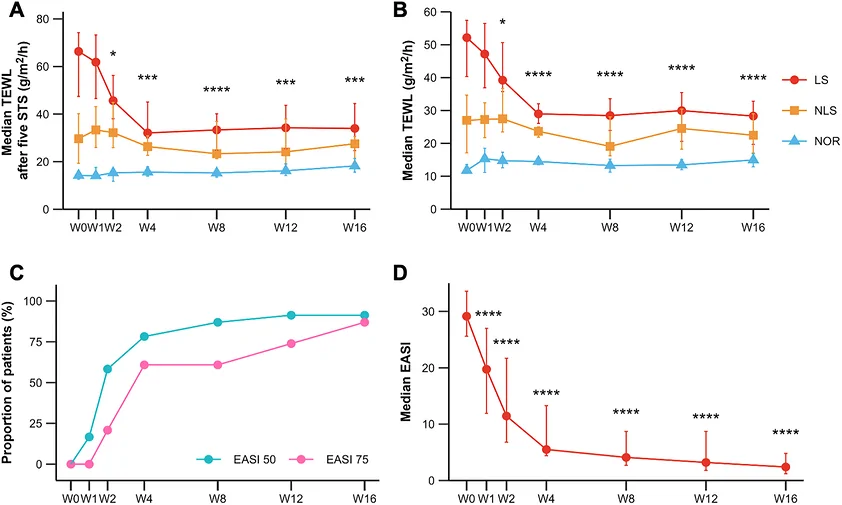
Dupilumab treatment improves TEWL measures and outcome. (A) Changes in TEWL measured after five consecutive tape strips in lesional AD, non‐lesional AD, and normal skin at each visit from baseline (W0) to W16. (B) Changes in TEWL in lesional AD, non‐lesional AD, and normal skin at each visit from W0 to W16. (C) Proportion of patients achieving EASI50 and EASI75 over 16 weeks of dupilumab treatment. (D) Changes in EASI scores in AD lesions from W0 to W16. Data are presented as medians with 95% CIs. Statistical comparisons were performed using a mixed‐effects model followed by Dunnett's multiple comparisons test. *p__adj_ < 0.05, **p__adj_ < 0.01, ***p__adj_ < 0.001, and ****p__adj_ < 0.0001 versus baseline. AD, atopic dermatitis; CI, confidence interval; EASI, Eczema Area and Severity Index; EASI50, 50% improvement in EASI score; EASI75, 75% improvement in EASI score; LS, lesional; NLS, non‐lesional; NOR, normal; STS, skin tape stripping; TEWL, transepidermal water loss; W, week.

### 2.3 Clinical Severity

The median (IQR) Eczema Area and Severity Index (EASI) scores significantly decreased from 29.2 (22.7–36.8) at baseline to 11.4 (6.8–21.9) at Week 2 (mean difference, 14.6; 95% CI, 11.0–18.2; adjusted *p* < 0.0001), and this reduction was maintained through Week 16 (Figure 2D). A 50% and 75% improvement in EASI score (EASI50 and EASI75) were achieved by 78.3% and 60.9% of patients at Week 4, respectively, and by 91.3% and 87.0% at Week 16 (Figure 2C). Scores for the pruritus Numerical Rating Scale (p‐NRS), SCORing Atopic Dermatitis (SCORAD), Patient‐Oriented Eczema Measure (POEM), and Dermatology Life Quality Index (DLQI)/Children's Dermatology Life Quality Index (CDLQI) showed significant reductions during the treatment period, whereas the sleep Numerical Rating Scale (s‐NRS) scores gradually increased (Figure S2).

### 2.4 Safety

Of the 24 patients with AD, 13 (54.2%) and 11 (45.8%) experienced 27 adverse events (AEs) and 20 treatment‐emergent AEs (TEAEs), respectively. All AEs reported during the study were mild‐to‐moderate in severity. Serious TEAEs, deaths, or dose adjustments were not observed (Table S1).

### 2.5 Proteomic Analysis of the Stratum Corneum

To further elucidate the proteomic alterations induced by IL‐4Rα blockade, a longitudinal proteomic analysis was conducted on 20 patients with AD receiving dupilumab and 17 healthy volunteers at baseline (Week 0) and Weeks 1, 2, 4, 8, 12, and 16. Samples with protein detection rates < 20% were excluded from the analysis. To minimize bias from missing values, only proteins quantified in ≥ 70% of both lesional and non‐lesional AD skin samples were retained. After quality control, 2223 proteins across 388 samples (lesional AD skin, *n* = 135; non‐lesional AD skin, *n* = 137; and normal skin, *n* = 116) were included in downstream analysis. The filtered data were log_10_‐transformed.

To obtain a comprehensive profile of proteomic changes and identify co‐expressed protein modules, we applied weighted correlation network analysis (WGCNA) to the proteomic dataset (*n* = 2223; Figure 3). This network analysis revealed six protein modules, representing co‐expression patterns in normal skin, lesional AD skin, and non‐lesional AD skin samples. Each module was assigned a distinct color based on module size, with protein module 4 (p‐M4) being the largest (881 proteins) and p‐M6 being the smallest (26 proteins). Gene Ontology (GO) enrichment analysis was performed to identify biological functions associated with each skin co‐expression network module. The two largest modules (Figure 3A) were associated with the GO terms “cytosolic ribosome” (p‐M3) and “secretory granule lumen” (p‐M4). The remaining modules were related to “vesicle lumen” (p‐M1), “cortical cytoskeleton” (p‐M2), “keratin filament” (p‐M5), and “methylosome” (p‐M6).

**FIGURE 3:**
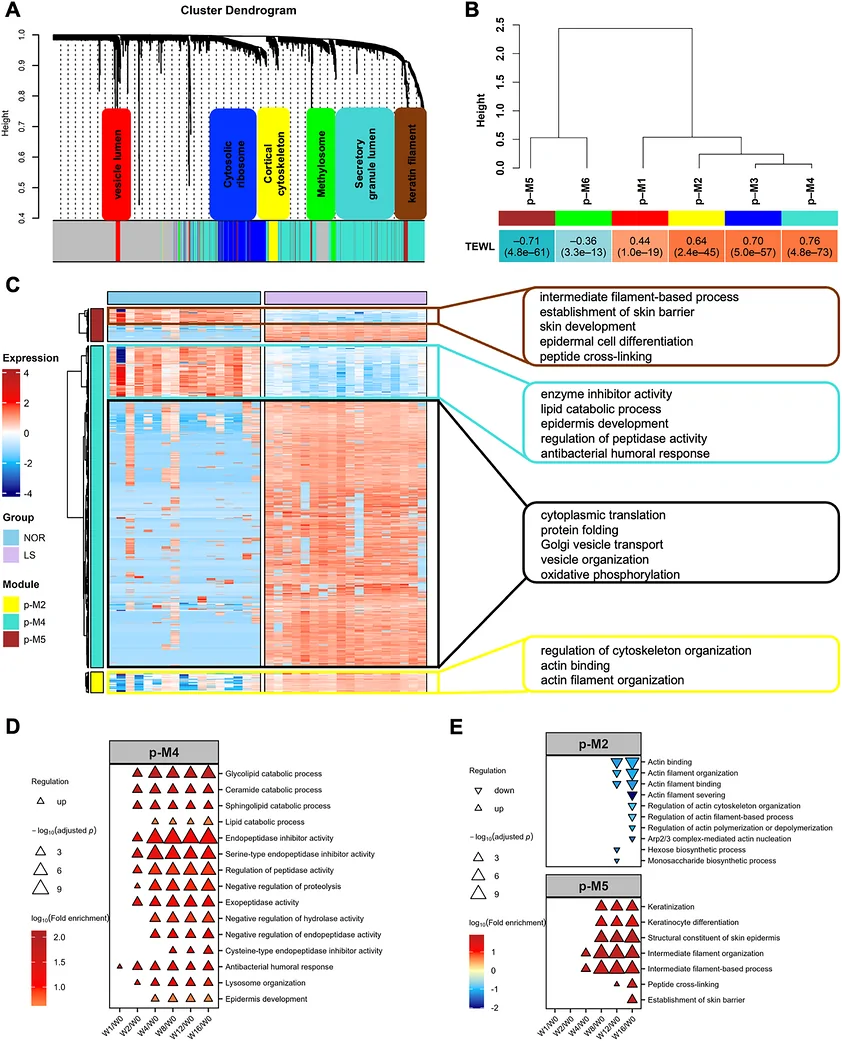
Protein co‐expression network of tape‐stripped human skin samples. (A) WGCNA dendrogram clustering 2223 proteins from lesional AD (*n* = 135), non‐lesional AD (*n* = 137), and normal (*n* = 116) skin samples into six distinct protein co‐expression modules (p‐M1 to p‐M6). Modules were annotated with Gene Ontology terms. (B) Pearson correlation between module eigengenes with TEWL. (C) Heatmap of proteins with differential abundance (adjusted *p* < 0.05 and |fold change| > 2) in the p‐M2, p‐M4, and p‐M5 modules at baseline (AD lesions vs. NOR skin). Gene Ontology analysis was performed for functional enrichment of differentially abundant proteins grouped into four boxes as shown. (D, E) Bubble plots showing Gene Ontology enrichment analysis of differentially abundant proteins in lesional AD skin at Weeks 1, 2, 4, 8, 12, and 16 versus baseline (W0). Enriched GO terms are for (D) the p‑M4 and (E) the p‑M2/p‑M5 modules. AD, atopic dermatitis; LS, lesional; NLS, non‐lesional; NOR, normal; p‐M, protein module; TEWL, transepidermal water loss; WGCNA, weighted correlation network analysis.

To assess the relationship between protein modules and skin barrier function, protein networks were correlated with TEWL (Figure 3B). Three modules—p‐M2 (*r* = 0.64, adjusted *p* = 2.4e−45), p‐M3 (*r* = 0.70, adjusted *p* = 5.0e−57), and p‐M4 (*r* = 0.76, adjusted *p* = 4.8e−73)—were positively correlated with TEWL. Notably, the secretory granule lumen (p‐M4) module exhibited the strongest correlation with TEWL (*r* = 0.76, adjusted *p* = 4.8e−73). Keratin filaments, which form part of the keratinocyte cytoskeleton, were abundant in the p‐M5 module and demonstrated a negative correlation with TEWL (*r* = −0.71, adjusted *p* = 4.8e−61). The GO terms for p‐M2, p‐M4, and p‐M5 were related to structural and secretory components of epidermal architecture; therefore, these three barrier‐relevant modules were selected for subsequent analyses.

At baseline, 754 proteins were significantly upregulated and 170 were significantly downregulated in AD lesions compared with normal skin (fold change ≥ 2). Dysregulated proteins within these modules were analyzed using hierarchical clustering and visualized in a heatmap (Figure 3C). Further pathway analysis revealed that signaling pathways linked to skin development and epidermal cell differentiation were downregulated (Figure 3C, brown box). GO enrichment analysis of proteins in the turquoise box identified GO terms related to the stratum granulosum (SG) and stratum corneum (SC). We focused on enriched terms related to skin barrier function, including lipid catabolic process, epidermis development, and antibacterial humoral response. In addition, pathway analysis identified increased enrichment of actin filament organization and actin binding pathways (Figure 3C, yellow box).

To explore the temporal patterns of pathway enrichment during dupilumab treatment, we compared biological functions across time points (Weeks 0, 1, 2, 4, 8, 12, and 16). In AD lesions, most lipid metabolism pathways exhibited increased enrichment scores during the first 2 weeks, coinciding with TEWL changes (Figure 3D). In non‐lesional AD skin, these changes became significant at Week 8 (Figure S3A). Furthermore, pathways associated with keratinocyte differentiation and keratinization showed increased enrichment in both lesional and non‐lesional AD skin at Week 8. In contrast, pathways governing actin binding, actin filament organization, and actin filament severing showed decreased enrichment in both lesional and non‐lesional AD skin by Week 16 (Figure 3E and Figure S3B).

Four skin‐barrier‑related functional categories were defined a priori using GO annotations: cell‐cell junction (GO:0005911), keratinization (GO:0031424), lipid metabolism (GO:0006629), and endopeptidase inhibitor (GO:0004866) [21, 22]. Among proteins in these categories, those whose abundance correlated strongly with TEWL are shown in Figure 4 (|*r*| > 0.6). In AD lesions, four lipid metabolism‐associated proteins—acid ceramidase (ASAH1), putative aminopeptidase PLBD2 (PLBD2), retinoid‐inducible serine carboxypeptidase (SCPEP1), and dehydrogenase/reductase SDR family member 6 (BDH2)—were negatively correlated with TEWL during dupilumab treatment (Figure S4). By Week 16, the abundance of 3'(2'),5’‐bisphosphate nucleotidase 1 (BPNT1), apoptosis regulator BAX (BAX), choline‐phosphate cytidylyltransferase (PCYT1A), and CD2‐associated protein (CD2AP) in AD lesions had significantly decreased from baseline (all adjusted *p* < 0.05; Figure 4). At baseline, the abundance of striatin (STRN), cingulin (CGN), and protein kinase C and casein kinase substrate in neurons 2 (PACSIN2) was significantly higher in AD lesions than in normal skin (all adjusted *p* < 0.05; Figure 4). By Week 16, the abundance of STRN, CGN, and PACSIN2 had decreased significantly from baseline and was no longer significantly different from that in normal skin (adjusted *p* = 0.14, 0.08, and 0.26 for STRN, CGN, and PACSIN2, respectively; Figure 4). Compared with normal skin at baseline, proteins essential for cornification, including desmocollin‐1 (DSC1), filaggrin‐2 (FLG2), caspase‐14 (CASP14), cystatin‐A (CSTA), cystatin‐M (CST6), and serpin B12 (SERPINB12), were downregulated in lesional AD skin (all adjusted *p* < 0.05; Figure S5).

**FIGURE 4:**
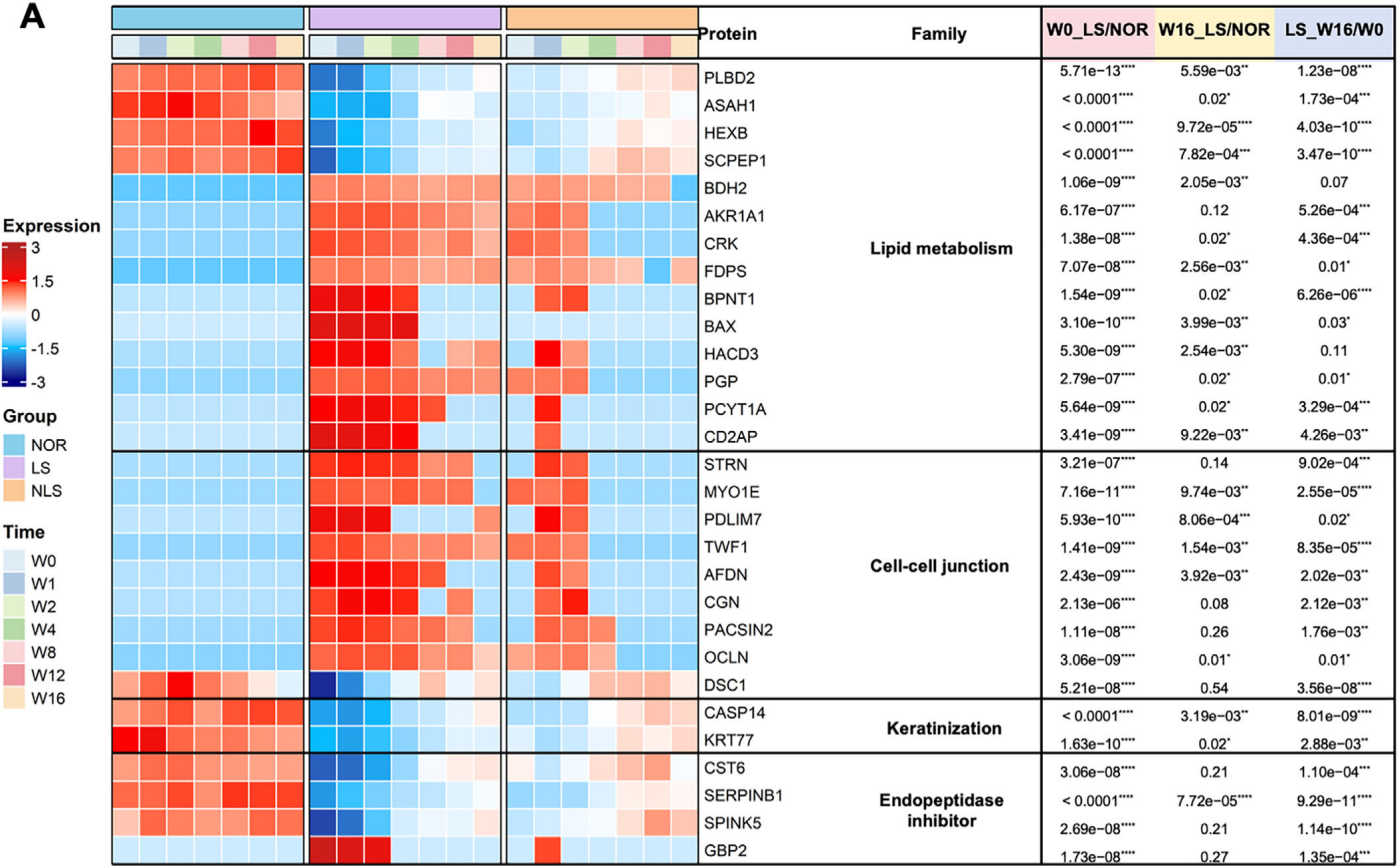
Longitudinal changes in skin barrier‐associated proteins. Heatmap of median skin barrier–associated protein abundance (cell‐cell junction, keratinization, lipid metabolism, and endopeptidase inhibitor) in lesional AD, non‐lesional AD, and normal skin over 16 weeks of dupilumab treatment. The accompanying table shows adjusted *p*‐values for AD lesions at W16 versus baseline (W0), and versus NOR skin at W0 and W16. Statistical significance was determined by ANOVA. *p__adj_ < 0.05, **p__adj_ < 0.01, ***p__adj_ < 0.001, and ****p__adj_ < 0.0001. AFDN, afadin; ASAH1, acid ceramidase; BAX, apoptosis regulator BAX; BDH2, dehydrogenase/reductase SDR family member 6; BPNT1, 3'(2'),5’‐bisphosphate nucleotidase 1; CASP14, caspase‐14; CD2AP, CD2‐associated protein; CI, confidence interval; CST6, cystatin‐M; DSC1, desmocollin‐1; FDPS, farnesyl diphosphate synthase; HACD3, very‐long‐chain (3R)‐3‐hydroxyacyl‐CoA dehydratase 3; HEXB, beta‐hexosaminidase; KRT77, keratin 77; LS, lesional; MYO1E, unconventional myosin‐Ie; NLS, non‐lesional; NOR, normal; OCLN, occludin; PCYT1A, choline‐phosphate cytidylyltransferase; PDLIM7, PDZ and LIM domain protein 7; PGP, phosphoglycolate phosphatase; PLBD2, putative aminopeptidase PLBD2; SCPEP1, retinoid‐inducible serine carboxypeptidase; SERPINB12, Serpin B12; TWF1, twinfilin‐1; W, week.

### 2.6 Lipidomic Analysis of the SC

To examine whether dupilumab treatment affects epidermal lipids in patients with AD, we performed untargeted lipidomics using liquid chromatography‐tandem mass spectrometry (LC‐MS/MS). A total of 396 skin samples (lesional AD, *n* = 140; non‐lesional AD, *n* = 140; and normal, *n* = 116) were collected at Weeks 0, 1, 2, 4, 8, 12, and 16. Overall, 611 lipid species from 36 subclasses were detected, including CERs, sphingomyelins (SMs), triacylglycerols (TGs), phosphatidylcholines (PCs), and others (Figure 5A). Among these, the TG subclass contained the highest number of lipid species (*n* = 159), while SM, zymosterol (Zy), and nine other subclasses contained only a single species. Next, we investigated the dysregulated lipids in AD lesions at specific time points compared with baseline. The results revealed dynamic changes in the lipidome throughout dupilumab treatment. Notably, t‐distributed stochastic neighbor embedding (t‐SNE) demonstrated that lipid profiles at Week 16 were distinctly separated from those at earlier time points, indicating a significant shift in lipid composition (Figure 5B).

**FIGURE 5:**
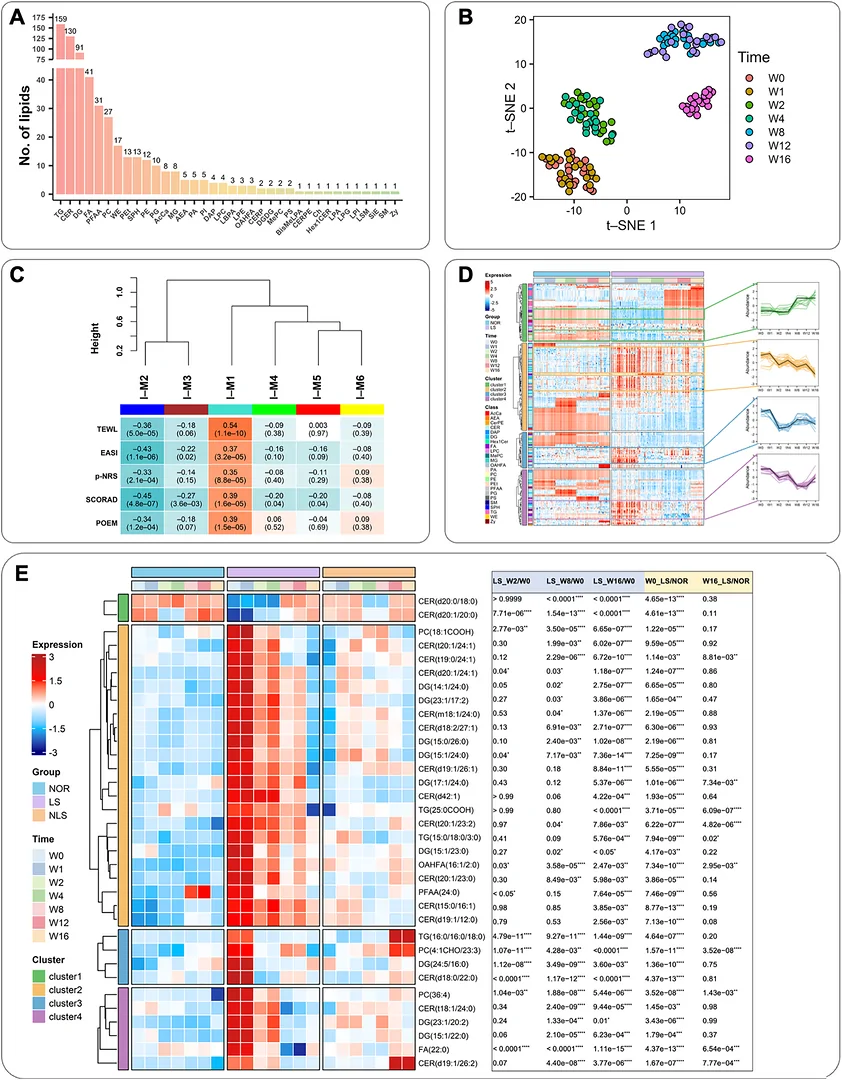
Lipidomic analysis of epidermal barrier‐associated lipids in AD lesions. (A) Number of lipid species detected in each subclass (total: 611 lipid species). (B) t‐SNE projection of 140 longitudinal lesional AD samples based on the levels of 611 lipid species. Each dot represents an individual sample, colored by sampling time point to visualize temporal shifts. (C) WGCNA clustering dendrogram of lipid species from longitudinal lesional AD samples (*n* = 140), identifying six lipid co‐expression modules (l‐M1 to l‐M6). The heatmap below shows the Pearson correlations between these modules and clinical measures (TEWL, EASI, SCORAD, p‐NRS, and POEM). (D) Heatmap of 238 lipids from l‑M1 and l‑M2 that differed significantly between lesional AD and normal skin at baseline (adjusted *p* < 0.05). These lipids were grouped into four temporal clusters using fuzzy c‐means clustering, followed by hierarchical clustering within each cluster. Highlighted boxes (Boxes 1–4) indicate lipid subsets whose post‐treatment profiles approached those of normal skin. (E) Heatmap of representative TEWL‐associated lipids from each box. Representative lipids were selected based on a Pearson correlation with TEWL (|*r*| > 0.4) and a ≥ 4‐fold difference between lesional AD and normal skin at baseline. The accompanying table shows adjusted *p*‐values for AD lesions at W2, W8, and W16 versus baseline (W0) and versus normal skin at W0 and W16. Statistical significance was assessed by ANOVA *p__adj_ < 0.05, **p__adj_ < 0.01, ***p__adj_ < 0.001, and ****p__adj_ < 0.0001. AcCa, acyl carnitine; AD, atopic dermatitis; AEA, anandamide; BisMeLPA, bis‐methyl phosphoglycerophosphate; CER, ceramide; CERP, ceramide phosphate; CERPE, ceramide phosphoethanolamine; Ch, cholesterol; DAP, dialkyl phthalate; DG, diacylglycerol; DGDG, digalactosyldiacylglycerol; EASI, Eczema Area and Severity Index; FA, fatty acid; Hex1Cer, hexosylceramide; LBPA, lysobisphosphatidic acid; l‐M, lipid module; LPA, lysophosphatidic acid; LPC, lysophosphatidylcholine; LPE, lysophosphatidylethanolamine; LPG, lysophosphatidylglycerol; LPI, lysophosphatidylinositol; LS, lesional; LSM, lysosphingomyelin; MePC, methyl phosphatidylcholine; MG, monoradylglycerol; NLS, non‐lesional; NOR, normal; OAHFA, (O‐acyl)‐ω‐hydroxy fatty acid; PA, glycerophosphoglycerophosphate; PC, phosphatidylcholine; PE, glycerophosphoethanolamine; PEt, plasmenylethanolamine; PFAA, polyhydroxyfatty acid amide; PG, glycerophosphoglycerol; PI, glycerophosphoinositol; POEM, Patient‐Oriented Eczema Measure; p‐NRS, pruritus Numerical Rating Scale; PS, glycerophosphoserine; SCORAD, SCORing Atopic Dermatitis; SiE, sitosterol ester; SM, sphingomyelin; SPH, sphingoid base; TEWL, transepidermal water loss; TG, triacylglycerol; W, week; WE, wax ester; Zy, zymosterol.

To identify the lipid species associated with clinical manifestations, we constructed a correlation network using WGCNA. Based on lipid profile similarity, six distinct lipid modules were observed in AD lesions (Figure 5C). Among them, lipid module 2 (l‐M2, *n* = 99) showed a negative correlation with TEWL (*r* = −0.36, adjusted *p* = 5.0e−05), SCORAD (*r* = −0.45, adjusted *p* = 4.8e−07), and p‐NRS (*r* = −0.33, adjusted *p* = 2.1e−04). In contrast, l‐M1 (*n* = 239) was positively correlated with TEWL (*r* = 0.54, adjusted *p* = 1.1e−10), SCORAD (*r* = 0.39, adjusted *p* = 1.6e−05), and p‐NRS (*r* = 0.35, adjusted *p* = 8.8e−05). These findings indicate that specific lipid modules strongly correlate with clinical severity, though their causal role in skin barrier properties or AD pathophysiology warrants further investigation.

Focusing on the specific modules identified above (l‐M1 and l‐M2), we selected 238 lipids exhibiting significant differences between AD lesions and normal skin at baseline (adjusted *p* < 0.05). To delineate their temporal trajectories during dupilumab treatment, these lipids were grouped into four distinct clusters using a fuzzy c‐means clustering approach. We then generated a heatmap employing hierarchical clustering within each cluster (Figure 5D). This visualization allowed us to visually identify specific lipid subsets (highlighted as “boxes”) whose post‐treatment lipid profiles appeared to normalize toward those of normal skin. Specifically, the lipids in Box 1 remained relatively stable until Week 4 and then increased progressively to a peak at Week 16. Boxes 2 and 3 showed a general downward trend with mid‐course fluctuations, including a transient rise in Box 3 during Weeks 4–8, followed by a further decline toward Week 16. Box 4 experienced a sustained decline from Weeks 0 to 12, reaching a nadir at Week 12, followed by a sharp rebound at Week 16. Next, we aimed to identify representative lipid species within each box by (1) analyzing the Pearson correlation coefficient (*r*) between lipid level and TEWL, filtering for |*r*| > 0.4, and (2) selecting lipid species that exhibited at least a four‐fold change between AD lesions and normal skin at baseline.

To visualize the temporal dynamics and post‐treatment normalization of these representative TEWL‐associated lipids, we generated a longitudinal heatmap comparing lesional AD, non‐lesional AD, and normal skin across all seven time points (Figure 5E). At baseline, lesional AD skin showed elevated levels of several neutral lipids (e.g., TGs and DGs) and certain CERs compared with normal skin (Figure 5E), suggesting altered lipid homeostasis. Longitudinal lipidome analysis over 16 weeks revealed distinct CER trajectory patterns. Specifically, a group of initially elevated CERs—including CER(t15:0/16:1), CER(d19:1/26:1), CER(d42:1), CER(t20:1/24:1), CER(d20:1/24:1), CER(m18:1/24:0), CER(d18:2/27:1), CER(t20:1/23:0), CER(d19:1/12:0), CER(d18:0/22:0), and CER(t18:1/24:0)—gradually decreased during treatment, and by Week 16, their levels did not differ significantly from those of normal skin. In contrast, CER(d20:0/18:0) and CER(d20:1/20:0) exhibited the opposite pattern. Both were significantly lower in lesional AD skin than in normal skin at baseline (adjusted *p* < 0.0001 for both), and increased progressively during dupilumab treatment. By Week 16, their levels no longer differed significantly from those in normal skin (adjusted *p* = 0.38 and adjusted *p* = 0.11, respectively). Furthermore, baseline elevations in specific acylglycerols—including DG(23:1/17:2), DG(14:1/24:0), DG(15:1/24:0), DG(15:0/26:0), DG(15:1/23:0), DG(24:5/16:0), DG(23:1/20:2), DG(15:1/22:0), and TG(16:0/16:0/18:0)—declined progressively during dupilumab treatment, and by Week 16, their levels did not differ significantly from those in normal skin (all adjusted *p* > 0.05).

Given that CERs' intermolecular interactions in model membrane systems are governed more by long‐chain base (LCB) length than by N‐acyl chain length [21], CER species were stratified primarily by LCB length, with acyl chain length retained as a secondary descriptor. We investigated whether the LCB composition of CERs correlated with TEWL (Figure 6 and Figure S6). In AD lesions, longitudinal TEWL were negatively correlated with total NDS(d16)‐CERs (*r* = −0.37, adjusted *p* = 1.7e−05), and positively correlated with total NP(t16)‐CERs (*r* = 0.48, adjusted *p* = 9.4e−09), total NP(t19)‐CERs (*r* = 0.57, adjusted *p* = 2.0e‐12), and total NH(t20)‐CERs (*r* = 0.46, adjusted *p* = 2.4e−08) during dupilumab treatment (Figure 6A). Similar correlation patterns were observed at baseline (Figure S6). These associations suggest that these lipid subgroups may be related to skin permeability barrier function, although causality cannot be inferred from correlational data. Compared with normal skin, the relative abundance of total NDS(d16)‐CERs was significantly decreased in AD lesions at baseline (mean difference, −0.8; 95% CI, −1.0 to −0.6; adjusted *p* < 0.0001), and returned to levels comparable to those in normal skin by Week 16 (mean difference, 0.1; 95% CI, −0.04 to 0.22; adjusted *p* > 0.05; Figure 6B). Moreover, the levels of total NP(t16)‐CERs and total NH(t20)‐CERs were significantly elevated in AD lesions, but rapidly decreased by Weeks 2 and 8, respectively (Figure 6B).

**FIGURE 6:**
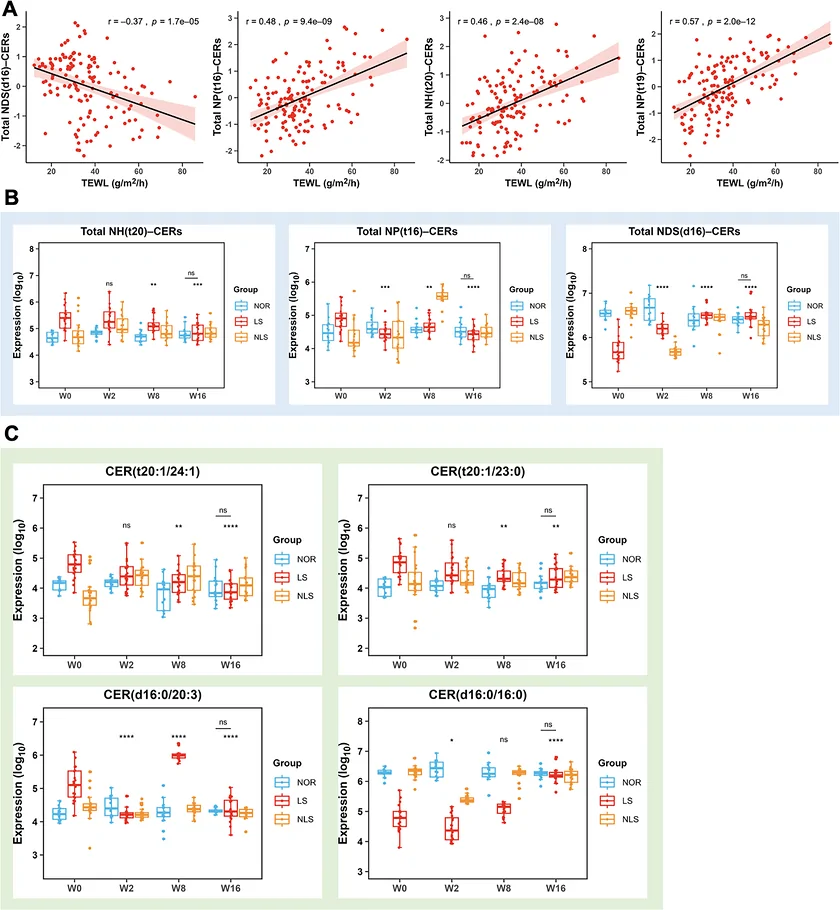
Dynamic changes in long‐chain base moiety of CERs during dupilumab treatment. (A) Pearson correlation between longitudinal changes in TEWL and relative abundance of total NDS(d16)‐CERs, total NP(t16)‐CERs, total NH(t20)‐CERs, and total NP(t19)‐CERs in AD lesions, respectively. Solid lines: linear regression fit; grey shading: 95% CIs. (B) Box plots showing changes in the relative abundance of total NDS(d16)‐CERs, NP(t16)‐CERs, and NH(t20)‐CERs at baseline (W0), W2, W8, and W16. Dots represent tape‐stripped samples from lesional AD, non‐lesional AD, and normal groups. (C) Box plots showing changes in CER(t20:1/23:0), CER(t20:1/24:1), CER(d16:0/20:3), and CER(d16:0/16:0) at W0, W2, W8, and W16. Dots represent tape‐stripped samples from lesional AD, non‐lesional AD, and normal groups. Statistical significance was determined by ANOVA. *p__adj_ < 0.05, **p__adj_ < 0.01, ***p__adj_ < 0.001, and ****p__adj_ < 0.0001. CER, ceramides; CI, confidence interval; LS, lesional; NLS, non‐lesional; NOR, normal; ns, not significant; TEWL, transepidermal water loss; W, week.

We next specifically examined the levels of individual CERs with distinct LCB compositions (t/d 16:0 and t20:1) during dupilumab treatment. At baseline, AD lesions showed significantly elevated levels of CER(d16:0/20:3), CER(t20:1/23:0), and CER(t20:1/24:1), whereas CER(d16:0/16:0) was significantly decreased compared with normal skin. Following dupilumab treatment, significant changes from baseline were observed for CER(d16:0/16:0), CER(d16:0/20:3), and CER(t20:1/24:1) by Week 2, and for CER(t20:1/23:0) by Week 8. By Week 16, the levels of all four ceramides normalized, with no significant differences remaining between AD lesions and normal skin (Figure 6C).

### 2.7 Multi‐Omic Changes in Response to Dupilumab Therapy

Across the 16‐week treatment period, 1074 proteins and 172 lipids showed a significant decrease in abundance compared with baseline, whereas 198 proteins and 118 lipids increased significantly (Figure 7A). A multi‐omics network analysis was performed using MetaboAnalyst to explore time‐dependent pathway changes in lesional AD skin during dupilumab treatment. Network analysis revealed that pathways related to sphingolipid metabolism exhibited increased enrichment at Week 2 in lesional AD skin (Figure 7B).

**FIGURE 7:**
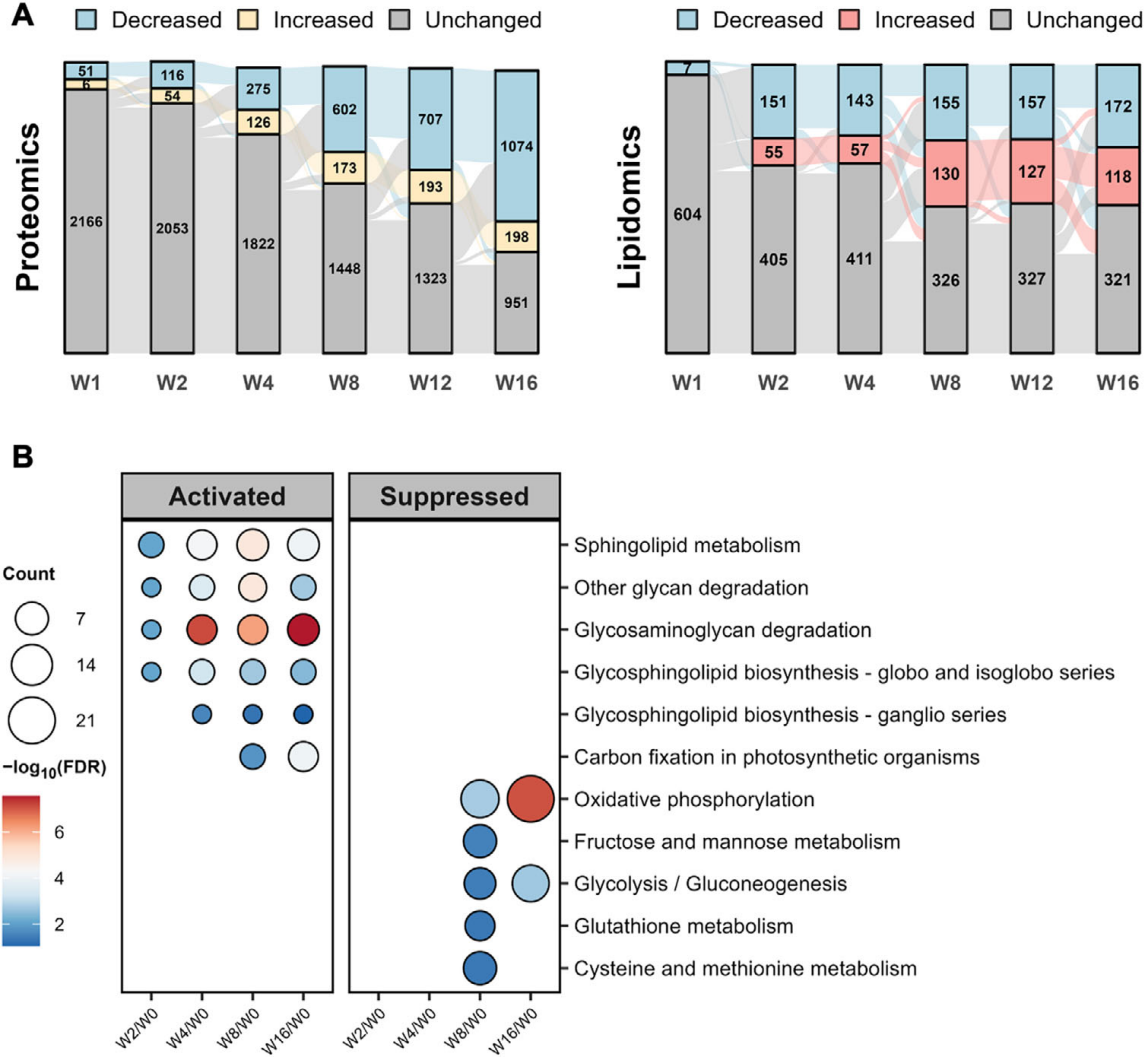
Multi‐omic changes in response to dupilumab treatment. (A) Numbers of proteins with differential abundance and lipids with differential levels at each time point versus baseline. (B) Enrichment network of proteins with differential abundance and lipids with differential levels at Week 16 versus baseline, generated using the MetaboAnalyst web tool. W, week.

## 3 Discussion

Because of the central role of IL‐4 and IL‐13 signaling in the pathogenesis of AD, dupilumab progressively improves clinical, molecular, and cutaneous abnormalities in patients with moderate‐to‐severe AD [23, 24, 25]. These cytokines suppress terminal differentiation and filaggrin expression even in the absence of *FLG* mutations [26, 27]. Together with barrier lipid abnormalities, they compromise both skin barrier integrity and antimicrobial function [8, 28]. Consistent with these observations, we found that dupilumab treatment for 16 weeks resulted in clinical and skin barrier function improvement, as demonstrated by decreased TEWL. Notably, although TEWL in lesional AD skin improved to levels comparable to non‐lesional AD skin by Week 16, it remained significantly higher than that of healthy controls. This trajectory suggests that while dupilumab facilitates barrier restoration, complete normalization may require prolonged therapy or could be constrained by the intrinsic subclinical barrier defects inherent to AD.

Our proteomic analysis provides a detailed characterization of protein networks linked to skin barrier function in AD, revealing dynamic changes across different stages of dupilumab treatment. Specifically, the secretory granule lumen (p‐M4) module exhibited the strongest positive correlation with TEWL. The SC is enriched in proteins involved in keratinization, lipid metabolism, and proteolysis, which are thought to contribute to barrier formation [29]. As keratinocytes differentiate from the spinous layer to the granular layer, lamellar bodies—lysosome‐related secretory organelles—fuse with the plasma membrane at the SG–SC interface, releasing lipid precursors, lipid‐processing enzymes, structural proteins, proteases, and antimicrobial peptides into the SC extracellular spaces [30, 31]. These secreted components contribute to the hydrophobic barrier that maintains skin homeostasis; in AD, impaired lamellar body secretion leads to defective lipid organization and barrier dysfunction [31, 32]. In our study, the p‐M4 module correlated with TEWL, consistent with previous studies showing that lamellar bodies contribute to barrier function [31] and desquamation [30]. We also observed a positive correlation between TEWL and the p‐M2 “cortical cytoskeleton” module in skin. This correlation suggests an association between the structural integrity of the skin and its barrier function. At baseline, the abundance of protein related to actin‐cytoskeleton was higher in lesional AD skin than in normal skin. The cytoskeleton and its intercellular junctions are thought to serve as a scaffold and rivets for maintaining epidermal structural integrity [33]. These changes may reflect chronic inflammation‐driven cytoskeletal remodeling, as demonstrated in other epithelial barriers [34], or altered actin dynamics that modulate AD severity [35]. Alternatively, a compensatory response to barrier defects cannot be excluded and requires direct testing.

Interestingly, our longitudinal proteomic profiling revealed a distinct temporal sequence of molecular events following IL‑4Rα blockade in AD lesions. We observed early enrichment of lipid metabolism pathways at Week 2, keratinocyte differentiation from Weeks 8 to 16, and decreased enrichment of actin cytoskeleton pathways from Weeks 12 to 16. A prior study demonstrated that Type 2 cytokines broadly alter lipid metabolism in the skin in ways that are detrimental to skin barrier function [28]. By blocking IL‐4/IL‐13 signaling, dupilumab treatment was associated with a rapid increase in lipid metabolic pathway enrichment (Figure 3), which coincided temporally with reduced TEWL at Week 2 (Figure 2). Given that keratinocyte differentiation and cytoskeletal reorganization are central to epidermal morphogenesis and homeostasis [33], this early enrichment of lipid metabolism‐related proteins may help establish conditions conducive to the subsequent structural restoration of the epidermal barrier.

At the ultrastructural level, the CE in AD is known to be deficient, with reduced corneocyte compaction and decreased intercellular lipids [9]. Our data demonstrated that dupilumab treatment selectively increased the abundance of multiple cornification‑associated proteins that were decreased at baseline. These results are consistent with dysregulation of genes related to keratinocyte differentiation reported in adult patients with AD [24]. Restoration of these cross‐linked, insoluble proteins is essential for rebuilding the CE and its associated cornified lipid envelope [36]. These structures, together with intercellular lipid lamellae, form the core of the skin permeability barrier [37]. Similarly, the decreased enrichment of actin cytoskeleton pathways from Weeks 12 to 16 is consistent with the resolution of inflammation‐driven cytoskeletal remodeling. These later changes may underpin the consolidation of epidermal architecture, given their central roles in epidermal homeostasis [33]. Supporting this, the elevated baseline levels of junctional (CGN) and cytoskeletal (PACSIN2, STRN) proteins normalized by Week 16 alongside improved barrier function, whereas reduced cornification proteins (e.g., DSC1) increased. Both patterns correlated with TEWL improvement, although further studies are needed to confirm their functional contributions.

SC lipids are essential for maintaining the epidermal permeability barrier [38]. In this study, t‐SNE analysis showed that lesional AD samples from different time points clustered separately, suggesting that the lipidomic signature dynamically shifts with inflammatory stage and treatment progression. The SC lipid matrix comprises a mixture of polar and non‐polar lipids, with CERs, non‐esterified FAs, and cholesterol as major components [39]. Sebum lipids include additional non‐polar lipids such as acylglycerols (TGs and DGs), FAs, wax esters, squalene, and cholesterol esters [40, 41]. Using WGCNA, we identified two lipid modules (l‐M1 and l‐M2) that were correlated with TEWL, suggesting that certain lipid species may serve as indicators of treatment response. Specifically, dupilumab treatment progressively restored initially depleted CER(d20:0/18:0) and CER(d20:1/20:0) while downregulating initially elevated lipids, including neutral lipids (e.g., DG(23:1/17:2), DG(14:1/24:0), and TG(16:0/16:0/18:0)) and specific CERs (e.g., CER(t20:1/24:1) and CER(t20:1/23:0)).

Impaired SC function in AD has been strongly associated with altered CER composition [42]. Structurally, CERs consist of a sphingoid base (also known as LCB) linked to a FA linked via an amide bond [43]. In mammals, five LCBs have been identified: dihydrosphingosine (DS), sphingosine (S), phytosphingosine (P), 6‐hydroxysphingosine (H), and 4,14‐sphingadiene (SD); each LCB differs in the number and position of hydroxyl groups and double bonds [44]. A previous study showed that lesional AD skin displays an altered CER composition, including increased α‐OH FA/sphingosine ceramide (CER[AS]) and short‐chain non‐OH FAs/sphingosine ceramide (CER[NS]) species (total carbon number C32‐39), together with reduced long‐chain CER[NS] (C51‐54), CER[NP], CER[NH], esterified ω‐hydroxy FA/sphingosine ceramide (CER[EOS]), CER[EOP], and CER[EOH] [45]. In this study, levels of total NDS(d16)‐CERs were negatively correlated with TEWL, whereas total NP(t19)‐, total NP(t20)‐, and total NH(t20)‐CERs showed positive correlation. These associations suggest that the LCB composition of CERs may be relevant to skin barrier function in AD. Further examination of individual CER species revealed a complex dysregulation pattern; among CERs containing t/d16:0 or t20:1 LCBs, CER(d16:0/16:0) was decreased in lesional AD skin at baseline, whereas CER(d16:0/20:3), CER(t20:1/23:0), and CER(t20:1/24:1) were increased. All of these abnormalities resolved by Week 16 of dupilumab treatment. While these correlative observations provide a basis for further investigation, the specific functional role of each CER species in AD remains to be elucidated.

In our multi‐omics analysis, pathway enrichment analysis revealed a distinct temporal pattern of sphingolipid metabolism activation—a key pathway for ceramide biosynthesis—with enrichment scores increasing as early as Week 2 in lesional AD skin. Notably, the early enrichment of sphingolipid metabolism pathways following IL‐4Rα blockade coincided with the observed reduction in TEWL. Integrated proteomic and lipidomic analyses showed that early sphingolipid metabolism pathway enrichment and specific ceramide changes at Week 2 preceded enrichment of epidermal differentiation pathways at Week 8, suggesting that early lipid‐related changes may facilitate subsequent epidermal structural repair. However, given the observational nature of this study, these temporal associations cannot definitively establish causality. Functional studies are required to directly test whether early lipid pathway activation drives barrier improvement.

This study has several limitations. The multi‐omic findings are primarily descriptive and correlative. Although we have identified multiple protein and lipid modules associated with skin barrier function, the observational nature of these data precludes the establishment of causality. Therefore, larger, multicenter, prospective studies with more diverse cohorts (including older and infant patients) and extended follow‐up periods are warranted to validate our observations and facilitate robust subgroup analyses. Furthermore, functional validation will be essential to elucidate the mechanistic links between the identified molecular alterations and improvements in skin barrier function.

## 4 Conclusion

This exploratory multi‐omics study delineates the temporal sequence of skin barrier recovery following IL‐4Rα blockade with dupilumab in patients with AD. Early enrichment of lipid metabolism pathways and changes in specific ceramide species at Week 2 coincided with reduced TEWL and preceded enrichment of epidermal differentiation–related pathways at Week 8, followed by gradual normalization of multiple dysregulated lipid species by Week 16. However, further functional studies are required to establish causality and to determine whether dupilumab regulates barrier function solely through IL‐4Rα blockade or also through reductions in specific inflammatory cytokines.

## 5 Materials and Methods

### 5.1 Study Design and Participants

The dupilumab BArrier function and LIpidomics Study in Atopic Dermatitis in China (BALISTAD‐CN) study was a Phase IV, open‐label, exploratory study (ClinicalTrials.gov identifier: NCT05624112) conducted Between November 25, 2022, and January 25, 2024. Twenty‐four patients with moderate‐to‐severe AD, diagnosed according to the Hanifin and Rajka criteria, were enrolled at the Dermatology Hospital, Southern Medical University (Guangzhou, China). Inclusion criteria were ages 12–65 years, an investigator's global assessment (IGA) score of ≥ 3, and presence of active lesions on the upper or lower extremities with IGA ≥ 2 at screening. Twenty age‐ and sex‐matched healthy volunteers, recruited through local community advertisements, were included as controls. Controls had no current or past skin or systemic diseases that could interfere with assessments.

This study followed the ethical principles of the Declaration of Helsinki. All participants provided written informed consent. For participants aged 12 to < 18 years, a legal guardian additionally signed an age‐specific informed consent form. The study protocol was approved by the Ethics Committee of the Dermatology Hospital, Southern Medical University (approval number: 2022094).

### 5.2 Treatment

Adult with AD received an initial subcutaneous dose of 600 mg of dupilumab (Sanofi, Paris, France), followed by 300 mg every 2 weeks (Q2W) through Week 14. Pediatric dosing was weight‑based: adolescents weighing < 60 kg received an initial subcutaneous dose of 400 mg followed by 200 mg Q2W; those weighing ≥ 60 kg received 600 mg followed by 300 mg Q2W.

### 5.3 Clinical Assessments and Sampling

The primary objective was to assess the change in TEWL after five STS procedures in lesional AD skin from baseline to Week 16 during dupilumab treatment. Secondary clinical assessments included the EASI score (range 0–72; higher scores indicate more severe disease), EASI50 and EASI75 response rates [46], and SCORAD scores (range 0–103) for assessing AD extent and severity [47]. POEM scores (range 0–28) were used to evaluate seven symptom frequencies over the past week (0 = no days, 4 = every day) [48]. HRQoL was evaluated using DLQI scores (range 0–30) for patients ≥ 16 years [49] and CDLQI scores (range 0–30) for children aged ≥ 4 to < 16 years [50], with higher scores reflecting poorer HRQoL. Itch intensity was measured using p‐NRS scores (range 0–10) and sleep quality using s‐NRS scores (range 0–10). Safety was also monitored. AEs were coded using Medical Dictionary for Regulatory Activities (MedDRA) version 21.1, and severity was assessed for AEs occurring in ≥ 1 participant.

TEWL was measured at each visit on both lesional and non‐lesional AD skin (upper or lower extremities, and the back) using a Tewameter TM300 (Courage + Khazaka Electronic, Köln, Germany) under controlled ambient conditions. Non‐lesional sites had to be ≥ 4 cm away from the index lesion. Before measurement, participants rested for ≥ 20 min at room temperature (19°C–23°C) with < 60% humidity. For matched healthy volunteers, measurements were performed at the corresponding anatomical sites. The final TEWL value was the average of three consecutive measurements. At each visit, 20 D‐Squame tape strips (22 mm diameter, 3.8 cm^2^; CuDerm, Dallas, TX, USA) were collected from representative lesional AD skin, non‐lesional AD skin, and normal skin, and stored at –80°C until processing. Each tape was applied to the skin for 10 s under standardized pressure (225 g/cm^2^) using a D‐Squame Pressure Instrument (D500) before being removed. For each sample, tape strips 1–5 were pooled for lipid extraction and strips 6–13 for protein extraction. The remaining strips (14–20) were not analyzed in the present study.

### 5.4 Proteomics

Samples were ground in liquid nitrogen, lysed in SDT buffer (100 mM NaCl) with 1/100 volume of DTT (Sigma, USA), and sonicated on ice for 5 min. After incubation at 95°C for 8–15 min and 2 min on ice, lysates were centrifuged at 12,000 *g* for 15 min at 4°C (D3024R, Scilogex). Supernatants were alkylated with sufficient iodoacetamide (Sigma, USA) for 1 h at room temperature in the dark, mixed with four volumes of pre‐cooled acetone (Beijing Chemical Factory), and incubated at –20°C for ≥ 2 h. Precipitates were collected (12,000 *g*, 15 min, 4°C), washed with 1 mL of cold acetone, and dissolved in DB buffer (8 M urea, 100 mM TEAB, pH 8.5).

Protein concentration was measured with the Bradford assay (Beyotime, China) using a BSA standard curve (0–0.5 g/L). For quality verification, 20 µg of protein per sample was resolved by 12% SDS‐PAGE and stained with Coomassie brilliant blue R‐250. Samples were adjusted to 100 µL with DB buffer and digested with mass spectrometry‐grade trypsin (Promega) at 37°C for 4 h, followed by additional trypsin and CaCl_2_ overnight. Formic acid (Thermo Fisher Scientific) was added to adjust the pH to < 3. After centrifugation (12,000 g, 5 min), supernatants were desalted on a C18 column, washed with 0.1% formic acid/3% acetonitrile, eluted with 0.1% formic acid/70% acetonitrile, and lyophilized.

Peptides were fractionated on a Waters BEH C18 column (4.6 × 250 mm, 5 µm) using a Rigol L3000 HPLC at 45°C into 4–6 fractions. A 4‐µg aliquot with 0.8 µL iRT reagent (Biognosys) was analyzed on an EASY‐nLC 1200 UHPLC coupled to a Q Exactive HF‐X mass spectrometer (Thermo Fisher) in DDA mode at Novogene Co. Ltd., using a home‐made analytical column (15 cm × 150 µm, 1.9 µm). MS1 scanned *m*/*z* 350–1500 at 120,000 resolution (AGC 3e6, 80 ms); the top 40 precursors were fragmented by HCD (MS2 15,000 resolution, AGC 5e4, 45 ms, NCE 27%, threshold 1.1e4, exclusion 20 s). DIA was performed on a Vanquish Neo UHPLC coupled to an Orbitrap Astral mass spectrometer (Thermo Fisher Scientific). Raw DIA data were processed with Spectronaut Pulsar (version 16; Biognosys AG, Schlieren, Switzerland) and searched against the *Homo sapiens* UniProtKB database (207,393 sequences downloaded on January 13, 2023) [51]. Search settings: automatic mass tolerance; fixed modification, cysteine alkylation; variable modifications, methionine oxidation and N‐terminal acetylation; and ≤ 2 missed cleavages. PSMs were filtered at 99% confidence, with 1% FDR at peptide and protein levels; retention times were calibrated with the iRT kit, with a precursor *Q*‐value cutoff of 0.01.

### 5.5 Lipidomics

Lipids were extracted using a modified methyl *tert*‐butyl ether (MTBE) method. To a 100 µL sample, 0.75 mL methanol was added in a glass tube with a Teflon‐lined cap, followed by the addition of a mixture of internal standards (PS(15:0/18:1)‐d7, LPE(18:1)‐d7, PA(15:0/18:1)‐d7, TG(15:0/15:0/18:1)‐d7, PE(15:0/18:1)‐d7, and PI(15:0/18:1)‐d7; Avanti Polar Lipids, USA). After vortexing, 2.5 mL MTBE was added and the mixture was shaken for 1 h at room temperature. After adding 0.625 mL MS‐grade water and incubating for 10 min, the sample was centrifuged at 1000 *g* for 10 min. The upper (organic) phase was collected, and the lower phase was re‐extracted with 1 mL MTBE/methanol/water (10:3:2.5, v/v/v). Combined organic phases were dried and reconstituted in 100 µL isopropanol. UHPLC‐MS/MS analyses were performed using a Vanquish UHPLC system (Thermo Fisher Scientific, Germany) coupled with an Orbitrap Q Exactive HF mass spectrometer (Thermo Fisher Scientific, Germany) at Novogene Co. Ltd. (Beijing, China). Separation was carried out on a Thermo Accucore C30 column (150 × 2.1 mm, 2.6 µm) at 40°C and 0.35 mL/min with a 20‐min gradient: 30% B at initial and 2 min; 43% B at 5 min; 55% B at 5.1 min; 70% B at 11 min; 99% B at 16 min; 30% B at 18.1 min. Buffer A: acetonitrile/water (6/4, v/v) with 10 mM ammonium acetate and 0.1% formic acid; buffer B: acetonitrile/isopropanol (1/9, v/v) with the same additives. MS operated in positive/negative modes: sheath gas 40 psi, sweep gas 0 L/min, auxiliary gas 10 L/min (7 negative), spray voltage 3.5 kV, capillary 320°C, heater 350°C, S‐Lens 50, scan 114–1700 *m*/*z*, AGC 3e6 (MS1)/2e5 (MS2), NCE 22/24/28 eV, injection 100 ms, isolation 1 *m*/*z*, and dynamic exclusion 6 s.

Raw data were processed with LipidSearch for peak alignment, peak picking, and quantitation (mass tolerance 5 ppm, retention time tolerance 0.05 min, and S/N 3). Peak intensities were normalized to the total spectral intensity.

### 5.6 Data Analysis and Statistical Analysis

Clinical endpoints were analyzed in the modified intention‑to‑treat population, which included all enrolled patients who received at least one dose of dupilumab (*n *= 24). Analyses were based on observed cases, using all available data up to the time of withdrawal or the last assessment without imputation for missing values. One patient withdrew at Week 3; thus, analyses from Week 4 onward included 23 patients with available data. Continuous variables were presented as mean ± standard deviation, median (IQR), or range (minimum–maximum), and categorical variables as counts and percentages. Changes in TEWL and clinical severity outcomes were assessed using a mixed‐effects model (a specific form of linear mixed model) in GraphPad Prism 8.0 (GraphPad Software, San Diego, CA, USA). Post‑hoc analyses for within‑group comparisons between baseline and each subsequent time point were performed using Dunnett's multiple comparisons test. Adjusted *p*‐values were reported, with *p* < 0.05 considered statistically significant.

For multi‐omic profiling, of the 44 enrolled participants (24 with AD and 20 healthy volunteers), six participants (three patients with AD and three healthy volunteers) from the initial pilot batch were excluded from all multi‐omic analyses to avoid potential batch effects and ensure data consistency. In addition, one additional patient who withdrew prior to Week 3 was excluded because of incomplete longitudinal tape‐stripped samples; this patient nevertheless contributed to the clinical modified intention‑to‑treat analysis up to the time of withdrawal. Consequently, the final analysis set for multi‐omics comprised 37 participants (20 patients with AD and 17 healthy volunteers). All proteomic and lipidomic analyses were performed using R software (version 4.3.2).

For t‐SNE, data were log_10_‐transformed and *z*‐scaled, then analyzed using the Rtsne package with the random seed set at 123 for reproducibility (v.0.17; perplexity = 20, θ = 0.05, and Truemax_iter = 1000). Correlations between lipid species and clinical scores were calculated using Pearson correlation coefficient via the “cor.test” function in R. The WGCNA R package (v.1.72‐5) was used to perform network analysis separately on protein samples (lesional AD skin [*n* = 135], non‐lesional AD skin [*n* = 137], and normal skin [*n* = 116]) and on lipid samples (lesional AD skin [*n* = 140]). The soft‑thresholding power was selected to achieve an approximate scale‑free topology (*R* ^2^ > 0.8).

Networks were constructed with the “blockwiseModules” function using average linkage hierarchical clustering of the topological overlap dissimilarity matrix. Modules were defined using the hybrid dynamic tree‑cut method: for lipidomics, power = 5, minModuleSize = 30, and deepSplit = 2; for proteomics, power = 12, minModuleSize = 20, and deepSplit = 4. Modules were merged at mergeCutHeight = 0.07, yielding six modules. Pearson correlation analysis between module eigengenes and clinical scores was performed using the “cor” and “corPvalueStudent” functions in R.

Temporal expression clustering of the lipidomic and proteomic data was performed using fuzzy C‑means (Mfuzz v.2.62.0). GO enrichment for proteins was performed with “enrichGO” in clusterProfiler (v.4.10.1) at high stringency, with the significance thresholds set as follows: pAdjustMethod = “BH,” pvalueCutoff = 0.05, and qvalueCutoff = 0.05. The input gene list consisted of proteins with differential abundance (|log_2_FC| > 1 and adjusted *p* < 0.05).

Skin‐barrier‐related protein categories were defined a priori using GO annotations: cell–cell junction (GO:0005911), keratinization (GO:0031424), lipid metabolic process (GO:0006629), and endopeptidase inhibitor activity (GO:0004866). Annotation sets for *Homo sapiens* were retrieved from AmiGO as direct annotations (annotations to child terms were not included) and matched to the quantified proteome by UniProt accession. Proteins assigned to more than one category were retained in all categories.

The multi‐omic network analysis was conducted using MetaboAnalyst v.6.0 (network analysis function) [52]. Differential abundance was assessed for each visit versus baseline in MetaboAnalyst (version 6.0; https://www.metaboanalyst.ca). Features with |log_2_FC| > 1 and FDR < 0.05 were classified as increased or decreased. At Weeks 2, 4, 8, and 16, these features were submitted to network analysis in MetaboAnalyst, mapping proteins (as gene symbols) and lipids (as compound names) onto biological interaction networks to explore their functional relationships. Lipids and proteins were mapped and visually explored in the KEGG global metabolic network (ko01100), and finally, we downloaded the network enrichment pathway results. Figures were generated in R (version 4.3.2) using ggplot2 (version 3.5.1). All multi‐omic analyses were considered exploratory and hypothesis‐generating. Reported correlations should not be interpreted as causal relationships.

## Author Contributions

**Taoming Liu**: formal analysis (equal), investigation (equal), project administration (supporting), writing – original draft preparation (lead). **Shuting Chen**: data curation (equal), formal analysis (equal), visualization (lead). **Weijia Zhao**: data curation (equal), formal analysis (equal). **Dake Di**: investigation (equal), formal analysis (supporting). **Jiao Liu**: formal analysis (equal). **Yuyang Tian**: investigation (equal). **Yurong Luo**: investigation (equal). **Yingping Xu**: resources (equal). **Ruzeng Xue**: resources (equal). **Li Ye**: resources (equal). **Xiaoping Pei**: investigation (supporting). **Bihui Yu**: investigation (supporting). **Yu Wang**: investigation (supporting). **Jiao Zhang**: investigation (supporting). **Xiaoling Yu**: investigation (supporting). **Wei Wen**: investigation (supporting). **Liyan Yuan**: investigation (supporting). **Suyun Ji**: investigation (supporting). **Claire Hu**: writing – review and editing (equal). **Dan Shen**: writing – review and editing (equal). **Yiqing Guo**: writing – review and editing (equal). **Yunsheng Liang**: conceptualization (lead), methodology (lead), supervision (lead), and writing – review and editing (lead). All authors have read and approved the final manuscript.

## Funding

This study was financially supported by Sanofi (China) Investment Co. Ltd., Shanghai Branch (LPS17250), and the National Natural Science Foundation of China (Grant No. NSFC82573980). Sanofi (China) Investment Co. Ltd. Shanghai Branch also provided financial support for medical writing assistance and open access fees. Sanofi contributed to the development of the study protocol with input from the corresponding author. Sanofi had no role in data collection, data analysis, data interpretation, manuscript preparation, or the decision to publish the manuscript. Sanofi provided funding for medical writing assistance; however, it had no role in the scientific content or revision of the manuscript. Data were collected by the study investigators and analyzed and interpreted by the academic authors. The first draft of the manuscript was written by the first author, with input from all co‐authors. The corresponding author had full access to all data and was responsible for the final decision to submit the manuscript.

## Ethics Statement

The study protocol was reviewed and approved by the Ethics Committee of the Dermatology Hospital, Southern Medical University (approval number: 2022094). The study was conducted in accordance with the Declaration of Helsinki. Written informed consent was obtained from all participants. For participants aged 12 to < 18 years, a legal guardian additionally signed an age‐specific informed consent form.

## Conflicts of Interest

Yunsheng Liang has acted as a speaker/consultant for AbbVie, Eli Lilly, LEO Pharma, Novartis, Pfizer, and Sanofi, and received investigator grants from Pfizer and Sanofi. Claire Hu, Dan Shen, and Yiqing Guo are employees of Sanofi. The other authors declare no conflicts of interest.

## Supporting information




**Supporting file**: mco271091‐sup‐0001‐SuppMat.docx

## Data Availability

The data supporting this study's findings are available from the corresponding author upon reasonable request. Raw omics data are not publicly available due to GDPR and other privacy regulations governing this Sanofi‐sponsored study. De‐identified processed data and clinical documents may be accessed by qualified researchers through the Sanofi data sharing platform, contingent upon a data sharing agreement and applicable data protection requirements. Further enquiries can be directed to the corresponding author.
