# Supplementary material for: Impact of Dupilumab Therapy on Skin Barrier Function in Patients With Moderate‐to‐Severe Atopic Dermatitis: A Phase IV, Non‐Randomized, Open‐Label, Parallel‐Group Clinical Study
Source: MedComm (2020). 2026 Oct 25;7(11):e71091. doi: 10.1002/mco2.71091 (PMC13695803; doi:10.1002/mco2.71091)
Supplement: Supplementary file 1 [file MCO2-7-e71091-s001.docx]

**Title:** **Impact of** **dupilumab therapy on** **skin barrier function in patients with moderate-to-severe** **atopic dermatitis: a phase IV, non-randomised, open-label, parallel-group clinical study**

**Running title:** Effect of dupilumab on skin barrier in AD

Authors: Taoming Liu^1,2#^; Shuting Chen^3,2#^; Weijia Zhao^3,2#^; Dake Di^4#^; Jiao Liu^2#^; Yuyang Tian^3,2^; Yurong Luo^3,2^; Yingping Xu^5^; Ruzeng Xue^2^; Li Ye^7^; Xiaoping Pei^2^; Bihui Yu^2^; Yu Wang^2^; Jiao Zhang^2^; Xiaoling Yu^2^; Wei Wen^2^; Liyan Yuan^2^; Suyun Ji^2^; Claire Hu^8†^; Dan Shen^9^; Yiqing Guo^10^; Yunsheng Liang^1,6,2*^

^#^Taoming Liu, Shuting Chen, Weijia Zhao, Dake Di and Jiao Liu contributed equally to this work.

^*^Corresponding author: Yunsheng Liang (yunshengliang@aliyun.com)

**Affiliations:** ^1^Hunan Key Laboratory of Medical Epigenomics, The Second Xiangya Hospital of Central South University, Changsha, China; ^2^Department of Dermatology, Dermatology Hospital, Southern Medical University, Guangzhou, China; ^3^The First School of Clinical Medicine, Southern Medical University, Guangzhou, China; ^4^Department of Traditional Chinese Medicine Dermatology, Dermatology Hospital, Southern Medical University, Guangzhou, China; ^5^Institute of Dermatology and Venereology, Dermatology Hospital, Southern Medical University, Guangzhou, China; ^6^Department of Dermatology, Second Xiangya Hospital, Central South University, Changsha, China; ^7^Evaluation Center for Skin Care Product Efficacy, Dermatology Hospital, Southern Medical University, Guangzhou, China; ^8†^Medical Affairs, Sanofi, Shanghai, China; ^9^Medical Affairs, Sanofi, Beijing, China; ^10^Medical Affairs, Sanofi, Guangzhou, China

^†^Formerly at Sanofi

**Corresponding author:**

Yunsheng Liang, M.D. & Ph.D.

Email: yunshengliang@aliyun.com

Telephone: +86-15973189908

Hunan Key Laboratory of Medical Epigenomics & Department of Dermatology, The Second Xiangya Hospital of Central South University, Changsha, China & Dermatology Hospital, Southern Medical University, Guangzhou, China.

# Supplementary Table

**Supplementary Table1.** Summary of safety in patients with atopic dermatitis

| **SOC**  **PT** | **Severity** | **Patients with AD (*n* = 24)** | |
| --- | --- | --- | --- |
|  |  | **Number of cases** | ***n* (%)** |
| At least one AE | Mild | 7 | 5 (20.8) |
|  | Moderate | 20 | 11 (45.8) |
|  | Severe | 0 | 0 |
| Infections and infestations | Mild | 3 | 3 (12.5) |
|  | Moderate | 8 | 7 (29.2) |
| Upper respiratory tract infection | Mild | 1 | 1 (4.2) |
|  | Moderate | 5 | 4 (16.7) |
| Conjunctivitis | Mild | 2 | 2 (8.3) |
|  | Moderate | 1 | 1 (4.2) |
| Hand-foot-and-mouth disease | Mild | 0 | 0 |
|  | Moderate | 1 | 1 (4.2) |
| Tonsillitis bacterial | Mild | 0 | 0 |
|  | Moderate | 1 | 1 (4.2) |
| Respiratory, thoracic and mediastinal disorders | Mild | 1 | 1 (4.2) |
|  | Moderate | 1 | 1 (4.2) |
| Cough | Mild | 1 | 1 (4.2) |
|  | Moderate | 1 | 1 (4.2) |
| Gastrointestinal disorders | Mild | 2 | 2 (8.3) |
|  | Moderate | 1 | 1 (4.2) |
| Abdominal pain | Mild | 2 | 2 (8.3) |
|  | Moderate | 0 | 0 |
| Gastritis | Mild | 0 | 0 |
|  | Moderate | 1 | 1 (4.2) |
| Skin and subcutaneous tissue disorders | Mild | 1 | 1 (4.2) |
|  | Moderate | 0 | 0 |
| Dermatitis atopic | Mild | 1 | 1 (4.2) |
|  | Moderate | 0 | 0 |
| Reproductive system and breast disorders | Mild | 0 | 0 |
|  | Moderate | 5 | 2 (8.3) |
| Dysmenorrhea | Mild | 0 | 0 |
|  | Moderate | 5 | 2 (8.3) |
| General disorders and administration site conditions | Mild | 0 | 0 |
|  | Moderate | 3 | 3 (12.5) |
| Pyrexia | Mild | 0 | 0 |
|  | Moderate | 2 | 2 (8.3) |
| Ill-defined disorder | Mild | 0 | 0 |
|  | Moderate | 1 | 1 (4.2) |
| Investigations | Mild | 0 | 0 |
|  | Moderate | 1 | 1 (4.2) |
| SARS-CoV-2 test positive | Mild | 0 | 0 |
|  | Moderate | 1 | 1 (4.2) |
| Injury, poisoning and procedural complications | Mild | 0 | 0 |
|  | Moderate | 1 | 1 (4.2) |
| Limb injury | Mild | 0 | 0 |
|  | Moderate | 1 | 1 (4.2) |

Abbreviations: AD, atopic dermatitis; AE, adverse events; PT, preferred term; SOC, system, organ, class.

# Supplementary Figure

Figure S1. Study Flow Diagram. AD, atopic dermatitis.

 Figure S2. Dupilumab treatment improves secondary outcome measures. Changes in (A) DLQI/CDLQI, (B) POEM, (C) SCORAD, (D) p-NRS, and (E) s-NRS scores in lesional AD skin from baseline (W0) to W16. Data are presented as medians with 95% CIs. Statistical comparisons were performed using a mixed-effects model followed by Dunnett’s multiple comparisons test. **p*__adj_ < 0.05, ***p*__adj_ < 0.05, ****p*__adj_ < 0.001, and *****p*__adj_ < 0.0001 versus baseline. Abbreviations: AD, atopic dermatitis; CDLQI, Children’s Dermatology Life Quality Index; CI, confidence interval; DLQI, Dermatology Life Quality Index; NRS, Numerical Rating Scale; POEM, Patient-Oriented Eczema Measure; p-NRS, pruritus Numerical Rating Scale; SCORAD, SCORing Atopic Dermatitis; s-NRS, sleep- Numerical Rating Scale; W, week.

 Figure S3. Bubble plots showing Gene Ontology enrichment analysis of proteins with differential abundance in non-lesional AD skin at Weeks 1, 2, 4, 8, 12, and 16 versus baseline (W0). Enriched Gene Ontology terms are for (A) the p‑M4 and (B) the p‑M2/p‑M5 modules. Abbreviations: AD, atopic dermatitis; p-M, protein module; W, week.

Figure S4. Correlations between epidermal barrier function-associated proteins and TEWL. Pearson correlations between TEWL and protein abundance related to (A) keratinization, (B) endopeptidase inhibitor, (C) cell-cell junction, and (D) lipid metabolism in lesional AD, non-lesional AD, and normal skin. Solid lines represent linear regression fit. Grey shading indicates 95% CIs. Abbreviations: AD, atopic dermatitis; AFDN, afadin; ASAH1, acid ceramidase; BAX, apoptosis regulator BAX; BDH2, dehydrogenase/reductase SDR family member 6; BPNT1, 3'(2'),5’-bisphosphate nucleotidase 1; CASP14, caspase-14; CD2AP, CD2-associated protein; CI, confidence interval; CST6, cystatin-M; DSC1, desmocollin-1; FDPS, farnesyl diphosphate synthase; HACD3, very-long-chain (3R)-3-hydroxyacyl-CoA dehydratase 3; HEXB, beta-hexosaminidase; KRT77, keratin 77; LS, lesional; MYO1E, unconventional myosin-Ie; NLS, non-lesional; NOR, normal; OCLN, occludin; PCYT1A, choline-phosphate cytidylyltransferase; PDLIM7, PDZ and LIM domain protein 7; PGP, phosphoglycolate phosphatase; PLBD2, putative aminopeptidase PLBD2; SCPEP1, retinoid-inducible serine carboxypeptidase; SERPINB12, Serpin B12; TEWL, transepidermal water loss; TWF1, twinfilin-1.

Figure S5. Longitudinal changes in cornification-associated proteins. (A) Heatmap showing the abundance of cornification–associated proteins in lesional AD, non-lesional AD, and normal skin over the 16-week period. Statistical significance was determined by ANOVA. **p*__adj_ < 0.05, ***p*__adj_ < 0.05, ****p*__adj_ < 0.001, and *****p*__adj_ < 0.0001. (B) Pearson correlations between TEWL and the abundance of CST6, FLG2, SERPINB12, DSC1, CSTA, and CASP14 in lesional AD, non-lesional AD, and normal skin. Solid lines represent linear regression fit. Grey shading indicates 95% CIs. Abbreviations: AD, atopic dermatitis; ANOVA, analysis of variance; CASP14, caspase-14; CI, confidence interval; CST6, cystatin-M; CSTA, cystatin-A; DSC1, desmocollin-1; FLG2, filaggrin-2; LS, lesional; NLS, non-lesional; NOR, normal; SERPINB12, serpin B12; TEWL, transepidermal water loss; W, week.

Figure 6S. Baseline correlations between long-chain base moieties of CERs and TEWL in skin. Pearson correlations between TEWL and the relative abundance of (A) total NDS(d16)-CERs, (B) total NH(t20)-CERs, (C) total NP(t16)-CERs, and (D) total NP(t19)-CERs at baseline in lesional AD, non-lesional AD, and normal skin. Solid lines: linear regression fit; grey shading: 95% confidence intervals. Abbreviations: AD, atopic dermatitis; CER, ceramide; LS, lesional; NLS, non-lesional; NOR, normal; TEWL, transepidermal water loss.
